# Residential Mobility and Breast Cancer in Marin County, California, USA

**DOI:** 10.3390/ijerph110100271

**Published:** 2013-12-23

**Authors:** Geoffrey M. Jacquez, Janice Barlow, Robert Rommel, Andy Kaufmann, Michael Rienti, Gillian AvRuskin, Jawaid Rasul

**Affiliations:** 1 BioMedware, Inc., 3526 West Liberty, Suite 100, Ann Arbor, MI 48103, USA; E-Mails: robert.rommel@biomedware.com (R.R.); andy.kaufmann@biomedware.com (A.K.); Gillian.avruskin@gmail.com (G.A.); jawaid.rasul@biomedware.com (J.R.); 2 Department of Geography, University at Buffalo—State University of New York, 105 Wilkeson Quad, Buffalo, NY 14261, USA; E-Mail: mvrienti@buffalo.edu; 3 Zero Breast Cancer, 4340 Redwood Highway, Suite C400, San Rafael, CA 94903, USA; E-Mail: janiceb@zerobreastcancer.org

**Keywords:** human mobility, space-time analysis, breast cancer, disease etiology

## Abstract

Marin County (California, USA) has among the highest incidences of breast cancer in the U.S. A previously conducted case-control study found eight significant risk factors in participants enrolled from 1997–1999. These included being premenopausal, never using birth control pills, lower highest lifetime body mass index, having four or more mammograms from 1990–1994, beginning drinking alcohol after age 21, drinking an average two or more alcoholic drinks per day, being in the highest quartile of pack-years of cigarette smoking, and being raised in an organized religion. Previously conducted surveys provided residential histories; while *Ǫ* statistic accounted for participants’ residential mobility, and assessed clustering of breast cancer cases relative to controls based on the known risk factors. These identified specific cases, places, and times of excess breast cancer risk. Analysis found significant global clustering of cases localized to specific residential histories and times. Much of the observed clustering occurred among participants who immigrated to Marin County. However, persistent case-clustering of greater than fifteen years duration was also detected. Significant case-clustering among long-term residents may indicate geographically localized risk factors not accounted for in the study design, as well as uncertainty and incompleteness in the acquired addresses. Other plausible explanations include environmental risk factors and cases tending to settle in specific areas. A biologically plausible exposure or risk factor has yet to be identified.

## 1. Introduction

Case-control studies (see Rothman *et al*. [1] for a discussion of case-control studies) have the substantial strength of being founded on carefully designed samples, giving them the power to evaluate whether observed health outcomes are associated with specific risk factors (the authors note that case-control studies are observational with attendant limitations in their scope of inference). Until recently, the unexplained risk—that not accounted for by the risk factors and covariates found significant in such studies—has not received much scrutiny. In recent years methods for allocating this unexplained risk to specific people, places, and times has received increased attention [2,3]. This may be accomplished within the framework of global and local clustering. Global clusters arise when there is statistically significant clustering of cases relative to controls when all of the cases and controls are considered simultaneously. Global clustering is usually assessed using a single statistic (called a global statistic) that assesses the presence of global clustering relative to a null hypothesis (e.g., spatial randomness). Local clusters describe an excess of cases relative to controls in specific portions of the study area [4]. This construct is used when explaining clustering of cases relative to controls in space and time.

This paper contributes to discourse focusing on the identification of unexplained risk factors affecting health outcomes in three ways. First, it examines breast cancer in Marin County, California, which has exhibited among the highest incidence rates of this condition in the United States for over a decade. The results of this study help to account for previously unexplained causes underlying the elevated risk for breast cancer in Marin County. Second, this study budgets the unexplained risk geographically and through time, using residential mobility data and accounting for known risk factors and covariates. While this does not evaluate alternative risk factors, the localization of unexplained risk can be a powerful tool in formulating causal hypotheses. Third, this study provides the first comprehensive demonstration of the method of *Ǫ* statistics, and fully demonstrates the approach using the Marin breast cancer data set. The overall objective of this study is to quantify and document the sources of unexplained risk for breast cancer in Marin County related to residential mobility of the study participants. This paper reports our findings for the evaluation of the following hypotheses:

*H1*: The cases and controls do not exhibit substantial residential mobility over the life course.
*Rationale*: The breast cancer risk factors found significant in the parent study operate at different points in a woman’s life course, some early in life, some later in life, and others involve long-term behaviors over several years. Substantial residential mobility in the study group suggests residence in Marin County may not be indicative of risk factors that occurred in Marin County.
*H2*: There is no statistically significant global clustering of breast cancer cases relative to controls after accounting for known risk factors and residential mobility.
*Rationale*: Global clustering might suggest the action of an unidentified risk factor not accounted for in the original case-control study design that impacts risk for most if not all of the cases (a large-scale signal, Global *Ǫ* statistic).
*H3*: There are no time periods when the breast cancer cases, considered as a group, exhibit statistically significant clustering relative to the controls.
*Rationale*: Large-scale spatial clustering at specific time periods may indicate past exposures that impacted many if not all of the cases (Global *Ǫ_t_* test).
*H4*: None of the cases exhibit statistically significant clustering over their life course, such that they tend to have other cases as neighbors.
*Rationale*: Clustering over the life course might indicate cases with similar residential histories—they either tend to travel together because of behavioral factors (e.g., seeking treatment, friendship) and/or have lived in areas that have elevated breast cancer risk (*Ǫ_i_* test).
*H5*: Cases that cluster over the life course (*Ǫ_i_*) are not part of local clusters at specific times (*Ǫ_t_*).
*Rationale*: Such a pattern (excess risk over the life course coupled with local clusters of excess risk) might indicate the action of ephemeral, geographically localized risk factors that were not accounted for in the parent case-control study.

## 2. Data

The data for this study come from a previous case-control study conducted by Wrensch *et al*. [5] that examined several recognized breast cancer risk factors and years of residence in Marin County, California. Cases were found among eligible female residents of Marin County who were diagnosed with breast cancer from 1997–1999; while women without breast cancer were recruited as controls through random digit dialing, and were frequency-matched by cases’ age at diagnosis and ethnicity. Subjects in both groups participated in either full in-person or abbreviated telephone interviews. Multivariate analyses determined that 285 cases were statistically significant as being more likely than 286 controls to report being premenopausal, never having used birth control pills, having a lower highest lifetime body mass index, having four or more mammograms from 1990–1994, beginning to consume alcohol after the age of 21, on average drinking two or more alcoholic drinks per day, being in the highest quartile of pack-years of cigarette smoking, and having been raised in an organized religion. Cases and controls did not significantly differ with regard to having a first-degree relative with breast cancer, a history of benign breast biopsy, previous radiation treatment, age at menarche, parity, use of hormone replacement therapy, age of first living in Marin County, or total years lived in Marin County. Results for several factors differed for women aged fewer than 50 years or 50 years and over. The study concluded that despite similar distributions of several known breast cancer risk factors, case-control differences in alcohol consumption suggest that risk in this high-risk population might be modifiable. The data used in the Wrensch *et al*. [5] study was obtained from Zero Breast Cancer, formerly Marin Breast Cancer Watch, which was the community principal investigator on the Adolescent Risk Factor Study and the Development of Breast Cancer in Marin County. Zero Breast Cancer also provided residential histories for the participants recruited via survey. These were then geocoded by the National Cancer Institute, with additional geocoding for place names and short street segments completed by BioMedware.

## 3. Methods

### 3.1. Space-Time Analysis

**Figure 1:**
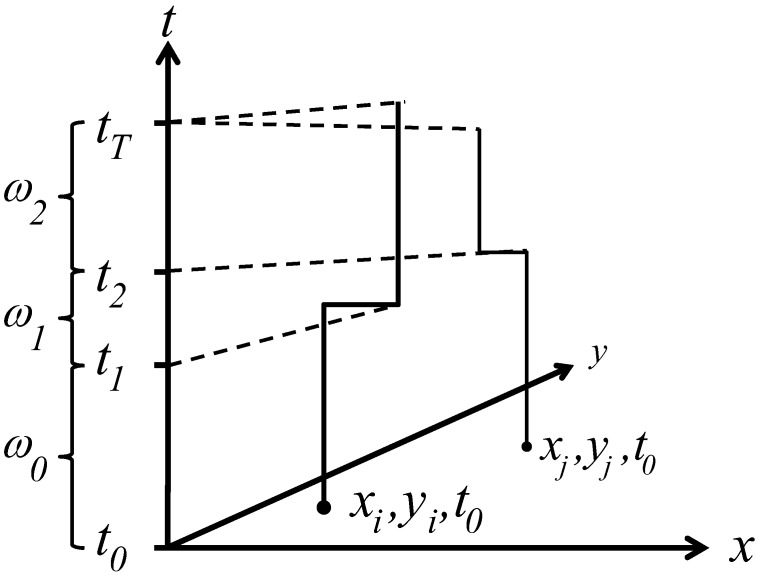
Residential histories as space-time step functions. The axes *x* and *y* define a geographic domain (*i.e.*, longitude and latitude decimal degrees), the *t* axes represents time (*i.e.*, date). The study extends from time *t_0_* to time *t_T_*. The residential histories for persons *i* and *j* are shown as step functions through space-time. For example, person *i* begins the study residing at location *x_i_, y_i_, t_0_*. They remain at that geographic coordinate until the instant before time *t*_1_, when they move to *x_i_, y_i_, t_1_*. The duration of time they reside at this first place of residence is *ω*_0_.

The data were loaded into BioMedware’s SpaceStat software, which represents residential histories as a space-time step function (Figure 1). This is a fundamentally different representation than that typically used with geographic data from case control studies as it represents places of residence as space-time threads (those locations where people lived throughout the study period—their residential history) rather than as spatial point distributions that are a disconnected set of points in space-time. The duration of place of residence is part of the data representation in Figure 1—how long a person resides at a given location clearly may impact their exposure [6], and therefore is incorporated into the underlying data model. Linked cartographic and statistical brushing of time-dynamic data on maps, histograms and time plots [7], was then used to quantify different aspects of residential mobility.

### 3.2. Ǫ-Statistics

*Ǫ*-statistics were calculated to identify local excesses of cases through space, time and space-time. Jacquez *et al*. [2,8] developed global and local tests for case-control clustering of residential histories. *Ǫ*-statistics rely on a matrix representation describing how spatial nearest neighbor relationships change through time. Time is defined by two approaches—duration-weighted and not duration-weighted. A person’s residential history is represented as a space-time thread using a step function (Figure 1). The not duration-weighted approach evaluates a given *Ǫ*-statistic whenever the geographic arrangement of the space-time threads changes—at times *t_0_, t_1_ …, t _T_*_‒1_ (refer to Figure 1). The duration-weighted statistics account for the corresponding duration of time (e.g., *ω*_0,_*ω*_1,_ …, *ω_T_*_‒1_) over which cases are obtained in a given geographic arrangement.

To identify the location and timing of significant clustering the following spatially and temporally local case-control cluster statistic is used:

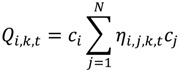
(1)

This quantity is the count, at time *t*, of the number of *k* nearest neighbors of case *i* that are cases, but not controls. Individuals *i* and *j* have case-control identifiers, *c_i_* and *c_j_*_,_ that are defined as 1 if a participant *i* is a case, and 0 otherwise. *N* is the total number of participants (cases and controls) in a study. The term *η_i,j,k,t_* is a binary spatial proximity metric that is 1 when participant *j* is a *k* nearest neighbor at time *t* of participant *i*; otherwise it is 0. Since a given individual *i* may have *k* unique nearest neighbors, the *Ǫ_i,k,t_* statistic is in the range 0..., *k*. When *i* is a control, *Ǫ_i,k,t_* = 0. When *i* is a case low values indicate cluster avoidance (e.g., a case surrounded by controls) and large values indicate a cluster of cases. When *Ǫ_i,k,t_* = *k* at time *t*, all of the *k* nearest neighbors of case *i* are cases. This statistic is recalculated for each participant every time there is a change in residence for person *i* or any of his/her *k* nearest neighbors. Therefore, Equation (1) reports a value for each residence at each and every time-geography of the residential histories (e.g., at times *t_0_, t_1_ …, t _T_*_‒1_ as shown in Figure 1). The user must specify the value for *k* before a statistic is calculated.

Duration-weighted statistics in which residences of longer duration are given greater importance are also used:

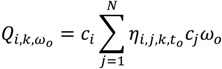
(2)

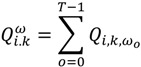
(3)

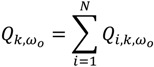
(4)

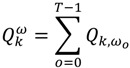
(5)


Equation (2) is similar to Equation (1), but is duration-weighted, as indicated by the notation *ω_0_*, which refers to the duration of time spent at a residence. The subscript “*o*” in *ω_0_* denotes the index for the duration *ω_0_ = t_0_*_+1_ − *t_0_*. Equation (3) is used to identify which cases are centers of spatial clusters through time; and is the sum, over all time durations, of Equation (2). Equation (4) is a global version and reports whether clustering occurs throughout the entire area at a particular duration (*ω_0_*) in time. It is calculated by summing Equation (2) over all cases at that particular duration in time. Equation (5), gives a measure of global case clustering of residential histories throughout the study area and over the entire study time period. It is calculated by summing Equation (4) over all *T*−1 durations *t*_0_, *t*_1_ …*, t_T‒_*_1_. This statistic indicates whether there is global clustering of residential histories when all of the residential histories over the entire study period are considered simultaneously. It is a measure of the persistence of global clustering, and is large when case clustering persists through time.

The unit of time used for inputting the data and for subsequent interpretation of the results is user-defined (*i.e.*, day, month, year,). The local *Ǫ*-statistics are therefore capable of detecting periodicities (*i.e.*, seasonal effects) that can occur when influenza outbreaks occur during fall and winter months; however, the temporal resolution of the time-unit must be fine enough to fully capture the effects of the periodicity of interest. For example, one cannot pick up seasonal effects using an annual time resolution. For these analyses, data was input with a temporal resolution of day, month, and year.

In this study *Ǫ_ikt_* is used to identify when and where an individual is the center of a local cluster. *Ǫ_ik_* identifies which individuals tend to be centers of clusters over their life-course, but not when those clusters occur. The global *Ǫ_k_* identifies whether global clustering tends to occur over the residential histories, but not when or where the clustering occurs.

Statistical significance was determined by randomizing the case-control identifiers over the residential histories under the null hypothesis of no association between places of residence and case-control status. Only case-control status was randomized, the integrity of the individual residential histories was maintained, and these were used to calculate the *Ǫ*-statistics. The randomization procedure was repeated over many iterations (999) to build up distributions of the *Ǫ*-statistics under the null hypothesis. The null hypothesis accounted for covariates and risk factors by employing the adjusted probabilities of being a case as calculated from logistic regression [8], which in this study were risk factors and covariates identified as significant by Wrensch *et al*. [5]. The range of possible *p*-values was determined by the number of randomizations applied to the null hypothesis. Given the computational power and time required for these analyses, 999 randomizations was the maximum reasonable number of iterations, generating a minimum *p*-value of 0.001.

#### 3.2.1. A Diagnostic Framework for *Ǫ*-Statistics in Relation to Disease Processes

In order to fully understand the utility of *Ǫ*-statistics in assessing specific etiologic hypotheses, it is necessary to understand the framework in which they are used. As an example, the three *Ǫ* statistic discussed above, *Ǫ_it_*—the local statistic, *Ǫ_i_*—the life-course statistic, and *Ǫ_t_*—the large-scale spatial cluster statistic, are evaluated here. The subscript *i* is a case identifier, so *Ǫ_it_* and *Ǫ_i_* make statements regarding clustering of individual cases. How might these be used to generate inferences regarding space-time cluster processes?

Assume a study population with *n* participants, with *n*_1_ that are cases and *n*_0_ that are controls. The beginning of the study period is *t* = 0, the end is *t* = *T*. Consider the sets defined as follows:

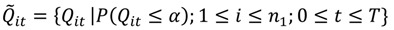
(6)

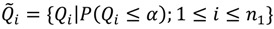
(7)

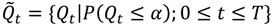
(8)

Here 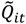 is the set of all *Ǫ_it_* that are statistically significant at the type I error level *α*, *Ǫ_i_*, 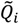 is the set of all *Ǫ_i_* that are statistically significant at *α*, and 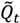 is the set of all *Ǫ_t_* that are statistically significant at *α*. It turns out that *Ǫ_t_* and *Ǫ_i_* are global statistics that assess case-clustering at specific times (e.g., *Ǫ_t_*) and over the life course of specific cases (e.g., *Ǫ_i_*) such that:

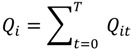
(9)

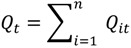
(10)

This means that the global statistics are the sums of the local statistics *Ǫ_it_*. This allows for the mapping of sets of the local statistics *Ǫ_it_* to sets of significant statistics 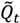 and 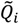. This mapping is comprised of those *Ǫ_it_* that contribute to the significant 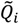 (through Equation (9)) and those *Ǫ_it_* that contribute to the significant 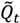 (through Equation (10)). Understanding this allows for the consideration of the following operations:

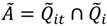
(11)

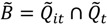
(12)

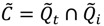
(13)

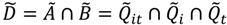
(14)


**Figure 2:**
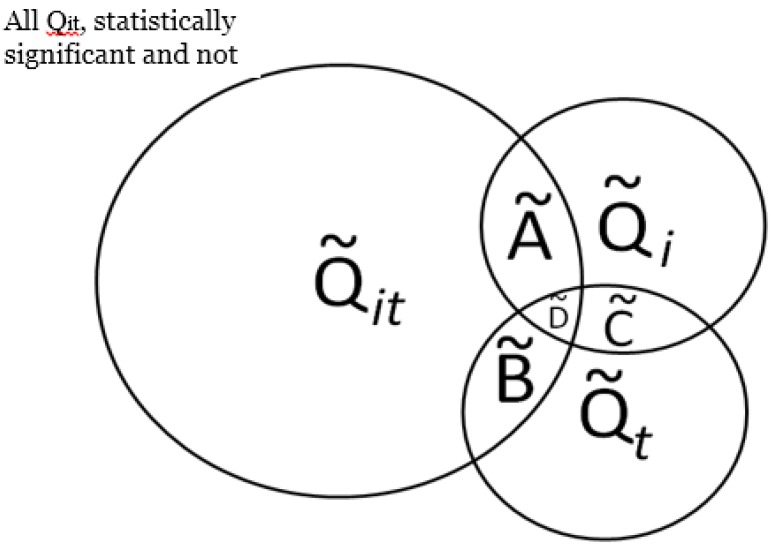
Venn diagram illustrating types of space-time clusters that can be identified using *Ǫ*-statistics. The rectangle represents all *Ǫ_it_* statistics in a study, significant or not. Each circle represents clusters that are found statistically significant locally (e.g., excess of cases about case *i* at time *t*, 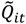), over a cases’ life course (e.g., excess of cases about the residential history of case *i*, 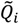), and globally at a given time *t* when all cases are considered together (e.g., large-scale spatial clusters at time *t*, 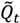). These cluster sets and their intersections (*Ã*, 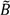, 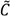, 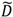) can provide insights into, and generate hypotheses regarding, disease etiologies. When the underlying *Ǫ*-statistics have been adjusted for the risk factors and covariates found significant in the parent case-control study these cluster types identify where, when, and to whom to allocate unexplained (e.g., excess) risk (Table 1).

The result of these set operations will be sets of the local statistics *Ǫ_it_* that contribute to the sets of significant global statistics that are the operands of Equations (11)–(14). These operations are represented in Figure 2.

#### 3.2.2. Assessing Overall Significance of Cluster Types

The cluster statistics within the cluster types defined by Equations (6)–(14) may be evaluated using the local space-time statistic *Ǫ_it_*, the life course statistic *Ǫ_i_,* and the spatial clustering statistic *Ǫ_t_*. The significance at a given *α* level yields membership in the sets illustrated in Figure 2. The number of local statistics in this case is quite large, and the use of the nominal type I error *α* will yield false positives. It is therefore necessary to derive statistical tests for evaluating the significance of the cluster types in Figure 2 that are not subject to erroneous inference attributable to multiple testing.

Each set corresponding to a cluster type as defined in Table 1 is comprised of significant local statistics. For example, recall that in Equation (1) 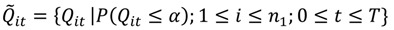. The number of elements in this set, 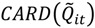, in a setting where true clustering exists, is comprised of both true positives and false positives (call this 
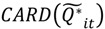
. If it is possible evaluate the probability of 
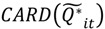
 under the null hypothesis of random labeling of the residential histories as cases or controls, and conditioned by the observed number of cases and controls, it is also possible to evaluate the significance of the cluster types in Figure 2 with a single test. In other words, the purpose is to assess the probability of observing the number of elements in the set 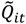. Table 2 enumerates the test statistics to be assessed, the cluster types they correspond to, and the probabilities to be evaluated.

**Table 1:** Description of cluster sets, summary space-time pattern descriptions, and disease etiologies that may give rise to those patterns.

Cluster set	Description	Pattern	Possible etiology
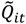	Cases (*i*) that at times *t* have a significant number of nearest neighbors that are cases	Cases (*i*) that at times *t* have a significant number of nearest neighbors that are cases.	*Infection*: Contagious process such that infection spreads from a case to its susceptible neighbors. Vector-borne disease process such that individuals in specific areas have increased risk of infection. *Chronic* (e.g., cancer): Increased cancer risk for individuals residing in local areas over a defined time period. Duration of elevated risk must be sufficiently long relative to the duration of time individuals live in the affected areas (e.g.,) exposure time must be sufficient to induce disease response.
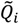	Clustering over the life course	Cases (*i*) who, over the study, have a significant number of nearest neighbors that are cases.	*Infection*: The “typhoid Mary” or “super-spreader” process, whereby case (*i*) (the super-spreader) is infectious over the study period and transmits infections to nearest neighbors. *Chronic* (e.g., cancer): A process whereby neighbors of case *i* have increased cancer risk and such risk is elevated over the life course of case *i*. An example would be behaviors that increase cancer risk for others such as second hand smoke. May also arise when groups with elevated risk tend to move or remain together over their life course (e.g., familial groups with common genetic and/or behavioral risk factors).
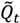	Temporal case clustering	Large scale spatial clustering of cases at time *t*. Clustering of cases relative to controls is significant at time *t* when all cases and controls are considered.	*Infection*: Infection outbreak such that the infection impacts a large portion of the study population; endemic phase of infection with multiple local outbreaks. *Chronic* (e.g., cancer): Chronic disease with an underlying infectious etiology (e.g., viral hypothesis of cancer) that impacts a large portion of the study participants; Disease risk mediated by environmental exposures that vary across the study area such that risk is elevated for a large number of study participants. Duration of elevated risk must be sufficiently long relative to the duration of time individuals live in the affected areas (e.g., exposure time must be sufficient to induce disease response).
*Ã*	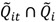	Locations and time when cases with significant clustering over their life course are members of a geographically localized cluster. Includes both ephemeral and persistent clusters.	*Infection*: Local foci of infection occurring at times *t* from which infected and infectious cases move away. *Chronic* (e.g., cancer): Local areas of persistent elevated risk that are sustained for a sufficient period of time that (1) disease risk is increased for individuals residing in the local area and (2) the duration of residence of cases in the area is of sufficient length to result in a significant *Ǫ_i_* statistic.
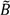	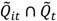	Cases (*i*) who, over the study, have a significant number of nearest neighbors that are cases.	*Infection*: Large-scale outbreak at specific times, *t*, that may be comprised of local pockets of infection. For vector-borne diseases this can arise when large portions of the study area have suitable vector habitat during some parts of the study period. *Chronic* (e.g., cancer): Large scale exposures that occur at a specific time(s) *t*. An example would be leukemia in response to the Chernobyl and Hiroshima incidents.
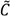	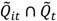	Cases that have clustering over their life course and are part of large-scale spatial clusters at times *t*. Includes cases whose *Ǫ_it_* are not statistically significant, and some whose *Ǫ_it_* are statistically significant.	*Infection*: Large-scale outbreak at times *t* with at least some of the resulting cases that (1) move together over their life course; and/or (2) remain infectious over their life course and continue to infect their neighbors. For a vector-borne disease this may arise when there is an initial large scale outbreak with some of the resulting cases continuing to be disease reservoirs (e.g., pathogen sources) whose infection can then be transmitted to neighbors. *Behavioral*: Individuals who have a behavior link that causes them to be at an increased exposure to an environmental factor, pathogen, or vector. The difference from *Ǫ_i_* is that here, the exposure factor must temporally “outbreak” in nature in that it either cycles in population like a vector/pathogen can (such as bird flu) or in severity for an environmental factor. For example, imagine a poultry reseller who moves around. He has an elevated risk any time a bird flu epidemic breaks out so will show a *Ǫ_i_* cluster and when the epidemics outbreak, there will be *Ǫ_t_* clusters. An example would be where a pesticide is applied but because of laws is phased out, but later on people start using it again. *Chronic* (e.g., cancer): Large scale exposures that occur at a specific time(s) *t* with some of the resulting cases that (1) move together through life course or (2) continue to reside in the affected area over most of the study period.
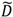	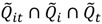	Cases that have clustering over their life course, are part of large scale clusters at time t and whose local clusters *Ǫ_it_* are all statistically significant.	Etiology is similar to set 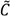 , but is restricted to include only those individuals that are centers of significant local clustering of cases at times *t*. For infection, this may be indicative of index cases; for chronic diseases this may indicate individuals who are within local pockets of the largest exposure.

**Table 2:** Statistics to evaluate the overall significance of the cluster types in a manner that does not involve multiple testing.

Cluster type	Cluster description	Test statistic	Probability of test statistic
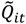	Local case-time	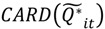	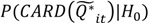
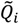	Life course	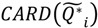	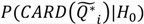
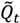	Temporal case clustering	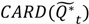	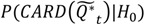
*Ã*	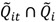	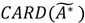	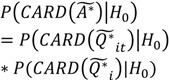
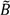	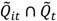	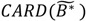	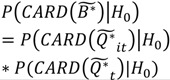
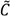	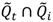	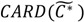	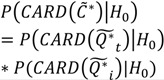
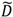	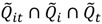	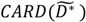	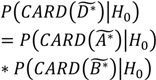

The predicted probabilities of the first 3 test statistics in Table 2 should follow a binomial probability. The general form of this probability is:

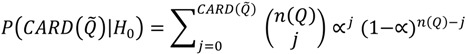
(15)

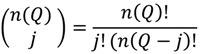
(16)

Here 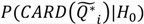 is the probability of the cluster set denoted 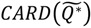 under the null hypothesis; these correspond to the entries in the column “probability of test statistic” in Table 2. 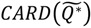 is the cluster set being considered; these are the entries in the column “Test statistic” in Table 2 and are the count of the number of significant clusters of that type. For example, recall that 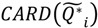 is the count of the number of observed cases that have significant clustering of cases about them over their life course. Here *n*(*Ǫ*) is the total number of occurrences of the statistic under consideration, whether significant or not. For example, *n*(*Ǫ_i_*) = *n*_1_, where *n_2_* is the number of cases in the study. Table 3 enumerates *n*(*Ǫ*) for the different cluster types. Finally, 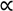 is the desired type I error of the test, often set to 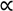 = 0.05.

**Table 3:** Number of possible test statistics including those significant and not significant for each cluster type, using the duration-weighted tests. Here *n*_1t_ is the number of cases extant in the study area at time *t*.

Cluster type	Cluster description	Test statistic	Number of possible elements in each set (*n*(*Ǫ*) in Equations (15) and (16))
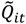	Local case-time	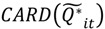	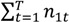
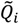	Life course	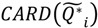	*n*_1*T*_
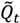	Temporal case clustering	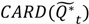	*T*

#### 3.2.3. Calculating the Empirical Type I Error under Multiple Testing

When simulating data so that there is no space-time case clustering, 
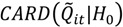
 is the number of false positives observed under the null hypothesis. The total number of possible tests as per Table 3 is 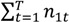, since the number of cases recorded in the data set will vary from one time to another, and since a local test is calculated for each case at each time point considered. The empirical type I error may then be estimated as:

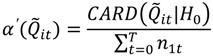
(17)

This can be calculated for 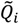 and 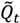 as:

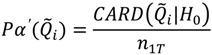
(18)

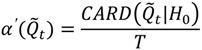
(19)


It is then possible to calculate the empirical type I error rate for a specific data set with a given conformation of residential histories, number of cases, and number of controls. Also, one can calculate the observed distribution of *p*-values ≤ 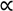 that are observed for a given set (say 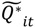) under the null hypothesis using a specific type I error (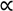) and number of simulation runs. This distribution is bounded on the right side by 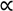, and on the left side by 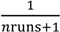, where *n*runs is the number of simulation runs conducted.

#### 3.2.4. Adjusting Critical Values of the Test for Different Cluster Types

When there are many cases and individuals are moving fairly often it is clear from Table 3 that the number of possible tests can become quite large, especially for the local test *Ǫ_it_*. One advantage of defining the cluster types described in Figure 2 is that the underlying tests are based on the number of elements in a set of a given cluster type, such as 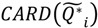. Should this statistic prove significant (e.g., 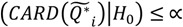), it is helpful to identify those Ǫ^*^_i_ subsumed within the set 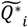 that are likewise significant. This is a much smaller number than the maximum number of *Ǫ_i_* that can be calculated, yet there is still a multiple testing issue.

A traditional multiple test correction factor could be used to control the family-wide error for each *Ǫ*-statistic, such as a Bonferroni correction or sequential methods like Simes-Hochberg or Hommel’s method [9,11]. However, these approaches tend to be conservative, especially for exploratory tests. Instead the authors control for the false discovery rate (FDR) [12]. This approach evaluates the fraction of false positives among all tests declared significant, controlling for family-wide type I error. Several variants of FDR approaches exist and it is necessary to choose an approach that allows us to optimally tune the threshold *p*-value used for significance to achieve the desired FDR [13]. This generates a *q*-value (no etymological relationship with *Ǫ*-statistics) for each *p*-value based on the overall distributions of *p*-values. The *q*-value represents the proportion of false positives among significant tests if this particular *p*-value is used as the significance threshold. Therefore, the choice of a critical *q*-value is determined by the desired FDR. An additional advantage is that this approach also estimates the total number of true positives (not just detected positives) in the family of tests, a measure of family-wide type II error. A traditional multiple tests correction adjustment is performed in order to adjust for significance of *Ǫ_i_*.

### 3.3. Analysis Steps

The following steps were used to sequentially evaluate the hypotheses:

1. *H1*: Evaluate residential mobility by mapping places of residence for the study participants at three spatial scales: Marin County, California, and the continental United States.
2. *H2*: Evaluate global clustering over the entire study using the Global *Ǫ*-statistic.
3. *H3*: Evaluate whether and when there are times that cases cluster relative to controls when all of the study participants are considered together using the *Ǫ_t_* statistic.
4. *H4*: Evaluate whether specific cases tend, over their life course, to have other neighbors as cases using the *Ǫ_i_* statistic.
5. *H5*: Using the significance of set *A*, evaluate whether cases that cluster over their life course (significant *Ǫ_i_*) are part of local clusters at specific times (*Ǫ_it_*).

## 4. Results

### 4.1. Geocoding

The original residential history database was comprised of 4,460 address records, an average of 7.81 addresses per participant. Many of these records did not have complete address information. The completeness of addresses was coded using “Number” (street name and number, a complete address), “Intersect” (street intersection), “Place” (place name, such as University of Wisconsin), “Street” (street name), “City” (City name), “State”, and “Country”. Our first pass attempted to geocode only the address records in the first two categories (“Number” and “Intersect”) that were in the U.S. There were 1,878 of these, or 42.11% of the total residential addresses. Of the 1,878 addresses, 1,568 were matched (83% were successfully geocoded, 35.16% of the total residential addresses). Additional geocoding efforts manually examined each of the 310 from the 1,878 that did not automatically geocode, and successfully located the majority of these addresses; however, the overall geocoding success rate was less than 40% of the total addresses. The geocoding match success rates were similar for cases and controls—38.19% for the former, and 38.13% for the latter.

### 4.2. Hypotheses

#### 4.2.1. H1: Cases and Controls Do not Exhibit Substantial Residential Mobility over the Life Course

The underlying rationale for H1 is that several of the breast cancer risk factors found significant in the parent study operate at different points in a woman’s life course—some early in life, some later in life, while others involve long-term behaviors over several years. Substantial residential mobility in the study group would suggest residence in Marin County may not be indicative of risk factors that occurred in Marin County. Evidence was found for substantial residential mobility in the study population leading to the rejection of the null hypothesis.

**Figure 3:**
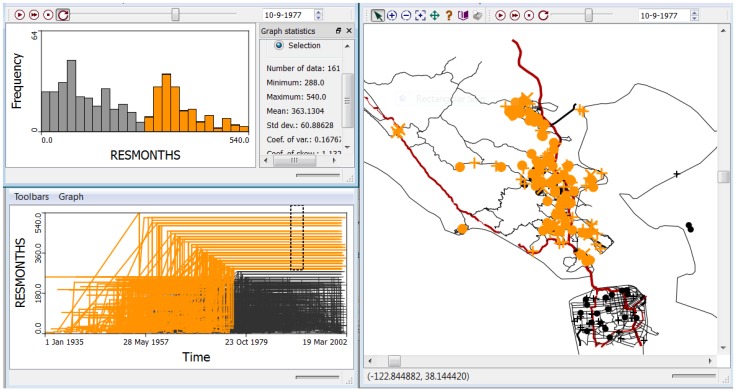
The study population is comprised of “movers” and “stayers”. Prior to 1980, the study population was comprised primarily of long-term residents, illustrated by the bimodal distribution of residence months (RESMONTHS, upper left histogram). The time plot (graph lower left) shows how residence time at each participant’s current residence changes through time. Each line corresponds to a study participant. Lines highlighted in orange are current Marin residents who have lived in the same home for at least 240 months (20 years), when the study was conducted. The map of Marin on 10-9-1977 (right) is comprised almost entirely of long-term residents.

First, the distribution of residence times (variable RESMONTHS) was examined for each study participant to assess time spent at their different residences as they moved from one place to another over the course of the study. The frequency distribution of residence times is temporally dynamic, and changes as the study participants move from one household to another. Two distinct subgroups were identified from this distribution—“movers” and “stayers” (see Figure 3).

**Figure 4:**
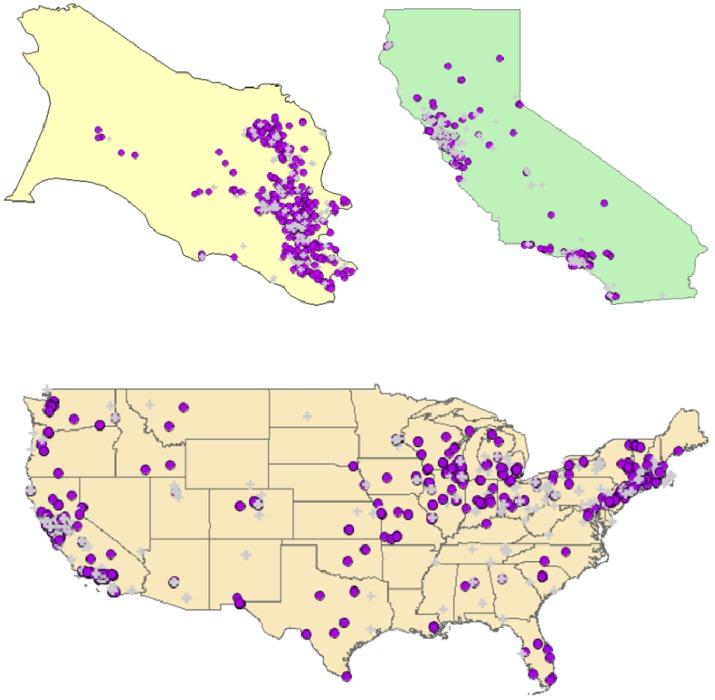
Life course place of residence for study participants in Marin County (**upper left**), California (**upper right**), and from across the United States (**bottom**). Cases are denoted as purple circles, and controls as gray crosses.

The strong bimodal distribution in Figure 3 identifies the “mover” and “stayer” sub-groups, with movers coming to Marin County from all portions of the United States. This raises an important question: How much of any observed clustering occurs among stayers (and hence may be attributable to local factors in Marin), and how much occurs among movers, and thus may represent risk factors that were imported from external populations (e.g., immigration of women from areas of high breast cancer risk such as Long Island)? To further quantify overall residential mobility in the study population places of residence for study participants were mapped at three spatial scales: Marin County, California and the Continental United States (see Figure 4).

#### 4.2.2. H2: There Is No Statistically Significant Global Clustering of Breast Cancer Cases Relative to Controls When Accounting for Known Risk Factors and Covariates and for Residential Mobility

This step evaluated the statistical significance of the global *Ǫ*-statistic (Equation (5)) to determine whether there was significant clustering of residential histories of cases when all of the cases and controls are considered over the entire study period. The existence of such global clustering might suggest the action of an unidentified risk factor, which is not accounted for in the original case-control study design, that impacts risk for most if not all of the cases (a large-scale signal from the Global *Ǫ*-statistic).

**Figure 5:**
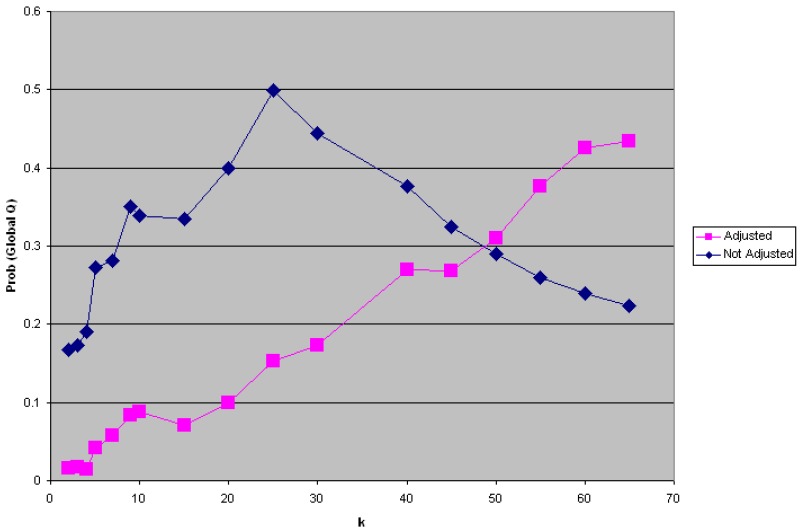
The sensitivity analysis using the Global *Ǫ-*statistic found significant global clustering of cases relative to controls after statistical adjustment for covariates and risk factors at *k* = 2, 3, and 4, with *p* < 0.01 at *k* = 4 nearest neighbors. This analysis was conducted at the spatial scale of Marin County.

The analyses were conducted by both adjusting and not adjusting for the risk factors and covariates found significant by [13]. There was no prior hypothesis regarding the spatial scale of effect, and it was necessary to conduct a sensitivity analysis to specification of *k*, the number of nearest neighbors considered. *k* = 2, 3, 4, 5, 7, 9, … up to 65 (Figure 5) were evaluated, and statistically significant global clustering was found after covariate adjustment with a minimum *p*-value < 0.01 at *k* = 4. The finding of significant global clustering indicates clustering of cases at the scale of the entire study period including all cases and controls. The next step is to localize the clusters to specific places and times.

One interesting, and unusual, feature of Figure 5 is that the probability of the global statistic after statistical adjustment for covariates is less than that for the unadjusted data at scales less than *k* = 50 nearest neighbors. When clustering is attributable to spatially structured covariates and risk factors (for example, demographic factors and socio-economic status are often similar at neighborhood levels) one often observes that adjustment for covariates reduces statistical significance.

This happens because the spatial structure in the health outcome is at least partly explained by the spatial structure in socio-demographic factors, and accounting for these factors reduces the significance of the observed clusters (e.g., they were explained by socio-economic clusters). Figure 5 demonstrates that statistical adjustment for covariates decreases the probability of global clustering such that the clustering of cases becomes more significant after adjustment for risk factors and covariate. It is as though a risk factor or covariate not accounted for in the parent case-control study design is unmasked once known risk factors and covariates are accounted for*.*

#### 4.2.3. H3: There Are No Time Periods When the Breast Cancer Cases, Considered as a Group, Exhibit Statistically Significant Clustering Relative to the Controls

This step identifies time periods with significant clustering of cases. Recall from H2, above, that significant global clustering over all participants and residential histories was statistically significant at *k* = 4. This step evaluates whether and when there are times that cases cluster relative to controls when all of the study participants are considered together using the *Ǫ_t_* statistic. The identification of large-scale spatial clustering at specific time periods may indicate past exposures that impacted many, if not all, of the cases. This is the specific pattern the *Ǫ_t_* statistic is designed to detect.

**Figure 6:**
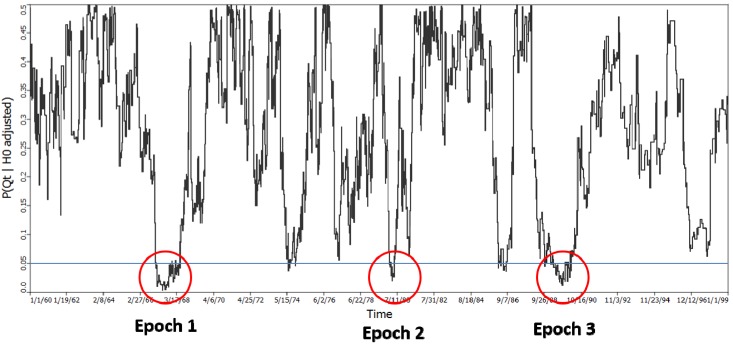
The probability of the *Ǫ_t_* test for spatial clustering of cases relative to controls through time. This test assesses whether and when there is spatial clustering of cases when all of the cases and controls are considered simultaneously at a given time *t*. *p* = 0.05 is shown by the blue horizontal line. The count of the number of observations below this line is 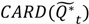 = 122 and is highly significant (*p* = 0.000001625).

The *Ǫ_t_* test defined by Equation (4) was performed at *k* = 4 using 999 randomization runs. This yielded one test statistic and *p*-value for each of the 1,568 time periods with unique geographic arrangements of cases and controls between 1-1-1960 and 1-1-1999 (Figure 6). Using a type I error of 0.05 it was expected that 1,568 × 0.05 = 78.4 of the global tests would be significant. 122 tests were identified with *p*-values less than 0.05, an excess of 43.6.

Equations (15) and (16) were used to evaluate the probability of observing 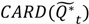 = 122, which yielded a probability of 0.000001625. This statistic is not subject to multiple testing, and indicates significant clustering of cases in some of the time periods considered. It does not however, identify when those time periods are, although they must be time periods from the set of 122.

When are those times with the strongest clustering of cases relative to controls—when the strength of clustering is defined by the most extreme (smallest) probabilities of *Ǫ_t_* under the null hypothesis of no clustering? To evaluate this question the probabilities of *Ǫ_t_* were ranked from smallest to highest, and then ordered through time. 3 epochs were identified as having significant clustering of cases relative to controls—February 1967 through February 1968 (Epoch 1); 1 April 1980 through 4 April 1980 (Epoch 2); and July 1989 through February 1990 (Epoch 3). Epoch 1 and epoch 2, which is relatively brief, were the focus of inquiries regarding the specific locations of case-clusters.

#### 4.2.4. H4: Cases Do not Exhibit Statistically Significant Clustering over Their Life Course

This step evaluates clustering over the life course, such that cases tend to have other cases as neighbors. The rationale underlying this hypothesis is that clustering over the life course may be indicative of cases from the study who (1) exhibit similar residential histories—they tend to travel together because of behavioral factors (*i.e.*, seeking treatment, friends or family relationship); (2) have lived in areas that have elevated breast cancer risk and then moved to Marin County; and (3) are long-term residents of Marin County from local areas with elevated breast cancer risk. This hypothesis was evaluated using the *Ǫ_i_* test in Equation (3) to identify individuals with clustering over their residential history, and Equation (7) to evaluate whether the observed number of significant *Ǫ_i_* (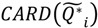) is itself statistically unusual. This second step has the advantage of not being subject to multiple testing, since only one measure (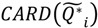) is evaluated The test for *Ǫ_i_* was performed using *k* = 5 and 999 randomization runs, while adjusting for the probability of being a case from the logistic regression in order to account for the risk factors and covariates found significant in the parent study by Wrensch *et al*. [5]. 30 cases were found to have significant clustering over their entire residential history. An evaluation of the probability of 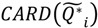 = 30 using the binomial expressions in Equations (15) and (16) yielded a probability of observing this outcome of *p* = 0.000115. 14.25 cases would have been expected to be significant at the nominal type I error of 0.05.

This analysis step identified those cases that had significant clustering over their life course.

#### 4.2.5. H5: Cases that Cluster over the Life Course (*Ǫ_i_*) Are not Part of Local Clusters at Specific Times (*Ǫ_it_*)

This step evaluates whether life course clusters form persistent local clusters. Such a pattern (excess risk over the life course coupled with local clusters of excess risk) might indicate the action of geographically localized risk factors that were not accounted for in the parent case-control study.

The evaluation of H4 identified 16 individuals with significant clustering over their life course. H4 identified two epochs—February 1967 through February 1968 (Epoch 1) and July 1989 through February 1990 (Epoch 3), both with significant large-scale spatial clustering of cases. Two types of participants were identified—movers, and stayers. The question then arises as to whether those cases with clustering over their life course spent a substantial portion of their life in Marin County. If they did, then a risk factor special to Marin County might be implicated. If they do not, then risk factors external to Marin may have contributed to the excess risk of breast cancer in Marin County.

**Figure 7:**
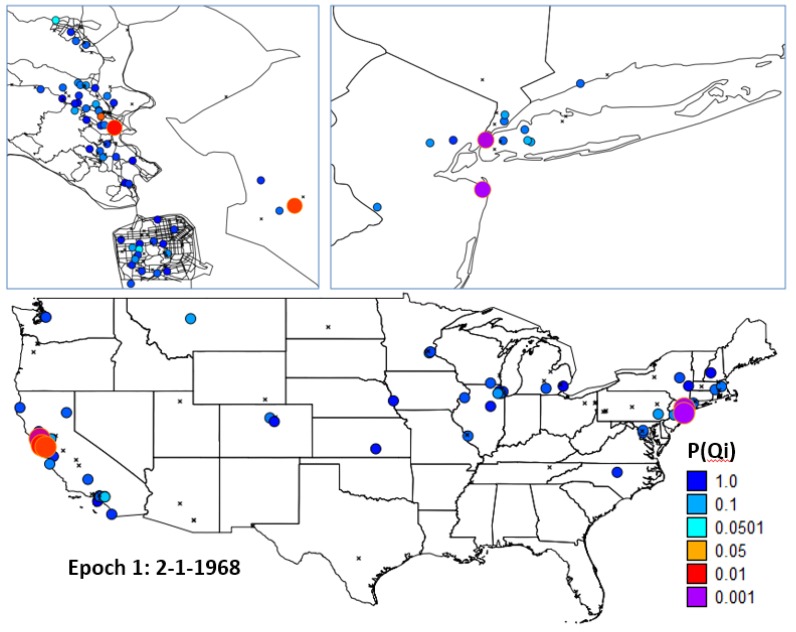
Life course clusters in Epoch 1. Locations of cases with significant clustering over their life course at the end of Epoch 1. Five cases had residential history data recorded on 2-1-1968. One case with significant clustering resided in Marin County (**upper left**), two were in the Bay area (**bottom**), and two in the Northeast near Long Island (**upper right**).

**Figure 8:**
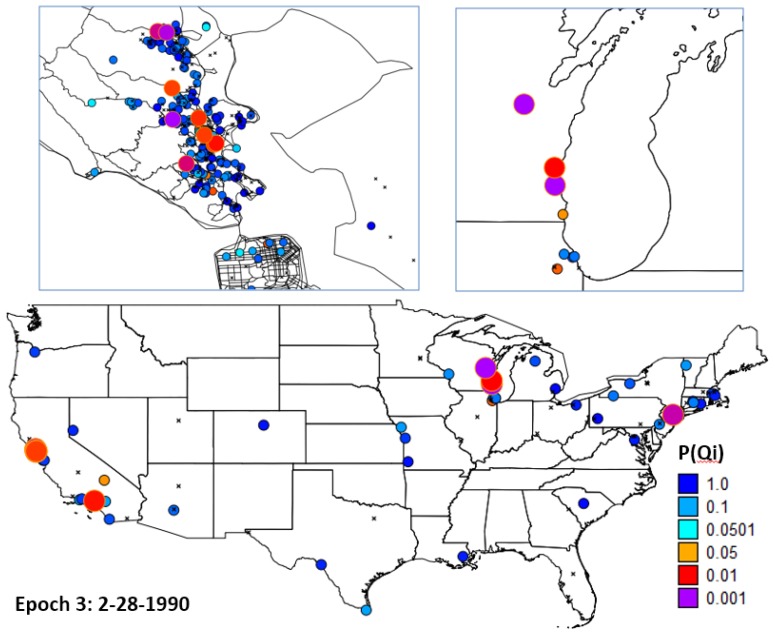
Locations of cases with significant clustering over their life course at the end of Epoch 3. At that time 9 cases with significant clustering resided in Marin County (**upper left**), with other clusters found in the upper Midwest (**upper right**), southern California and Long Island (**bottom**).

To address this question residential histories of the cases and controls were mapped, and the 16 cases with the smallest *p*-values for *Ǫ_i_* (life course clustering) were identified. Visual examination of the geographic distribution of these 16 cases was performed focusing on epochs 1 (Figure 7) and 3 (Figure 8).

**Figure 9:**
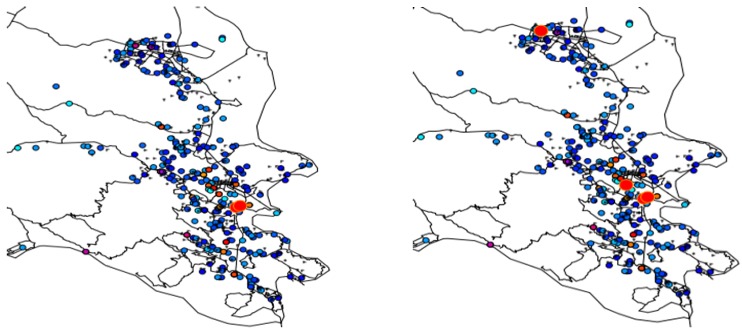
Local clusters of breast cancer cases (large red circles) for twenty year (**left**) and fifteen year (**right**) residents of Marin County, California. The observed clustering is statistically significant when residential mobility and significant risk factors and covariates are accounted for.

Many of the cases that comprised the study group in Marin County for the study by Wrensh *et al*. [5] were not life-time residents of the County. A portion of this group is quite mobile and it appears that many of the cases that contributed to clustering over the life course resided in parts of the Northeast, upper Midwest, and in portions of California outside of Marin County before settling in Marin. Figure 9 shows residency months of the study participants through time, with those 16 most likely life course clusters highlighted.

### 4.3. Synopsis and Synthesis: Locations of Persistent Life Course Clusters

Finally, one might ask where are cases that (1) have significant clustering over the life course; and (2) have been living in their current residence in Marin for fifteen and twenty years or more at the time the study period was conducted. Fifteen and twenty were selected as durations in years to evaluate how the locations of long-term clusters might change as a function of residence duration.

To address this question a scatterplot of the *p*-values of *Ǫ_i_* *versus* residence time at current residence during the study was constructed (1-1-1999), and from this individuals were identified that (1) are the most likely life-course clusters; and (2) have been at their current residence for at least 15 and also for 20 years. These cases were then mapped in Marin County (Figure 9). 

These are the places of residence for long-term residents of Marin County found to be statistically significant cluster centers after adjustment for the risk factors and covariates found significant by Wrensch *et al*. [5] These clusters are locations where excess breast cancer risk could plausibly be explained by unidentified, persistent environmental risk factors unique to Marin County. These could also be explained by a “case attractor” hypothesis where cases coming into Marin County tend to first reside in areas near these cluster foci. Behaviors where family members follow a “pioneer” to Marin, and in which the family has increased breast cancer risk due to behavioral or genetic factors would be consistent with the case attractor hypothesis. This also might arise when women with breast cancer move to be near a treatment center.

## 5. Discussion

This study identified three persistent clusters of breast cancer in Marin County that cannot be explained by known risk factors and covariates. There were defined by statistically significant clustering of cases over the residential histories of women who were residents of Marin County for at least fifteen years.

These clusters were defined using a statistic called *Ǫ_i_*, which evaluates the number of nearest neighbors of a breast cancer case that also were breast cancer cases, and not controls. Data on 285 cases were used in this study, of which 30 had probabilities of *Ǫ_i_* that were less than the type I error level of 0.05. This outcome (30 significant of 285 cases) was evaluated using a binomial probability, and it is considered a highly unlikely outcome (*p* = 0.000115). The authors concluded there is evidence of statistically significant clustering over the life course. Of these 30 significant cases, at the 0.05 level, we would expect to find 14.25 cases significant. The authors therefore continued to analyze only those remaining 16 cases with the smallest *p*-values, once multiple testing was accounted for.

After adjustment for known risk factors and covariates significant global clustering of cases relative to controls was also found using the global *Ǫ*-statistic (*p* < 0.01). This approach considers all cases and controls through time, yields one test statistic, and is not subject to multiple testing.

Further statistically significant clustering was identified using the *Ǫ_t_* statistic, which evaluates clustering of cases relative to controls at specific time points. Of the 1,568 time periods that were defined between 1-1-1960 and 1-1-1999 whenever a woman moved from one residence to another, 122 had significant *Ǫ_t_* statistics. This count (122) is a highly unlikely outcome (*p* = 0.000001625). Of these 122 periods, one would expect at the 0.05 level to find 78.4 to be statistically significant under the null hypothesis. The remaining 44 most extreme cases defined three time periods (Epochs) when there was significant clustering of cases relative to controls when all of the study participants are considered simultaneously. These are February 1967 through February 1968 (Epoch 1), 1 April 1980 through 4 April 1980 (Epoch 2), and July 1989 through February 1990 (Epoch 3). Epoch 2 is brief and focus was directed the specific locations of case-clusters to Epochs 1 and 2.

The Marin study population is comprised of “movers” and “stayers”, and a substantial number of the cases the authors identified with significant clustering over their residential histories originated from outside Marin County, coming from New York near Long Island, the upper central Midwest, and other parts of California. It is possible those cases identified as “movers”, especially those coming from known areas of high breast cancer risk such as the New York area, may have been impacted by environmental, behavioral and/or, genetic risk factors associated with those outside areas. Specifically, their breast cancer risk was less likely to have been influenced by factors that would have been unique to Marin County since their cumulative residence time in Marin County was shorter than for long-term residents.

The finding of significant clustering of breast cancer cases among long-term residents may indicate the role of geographically localized risk factors not accounted for in the parent case-control study design. Plausible hypotheses include environmental risk factors, differential migration such that cases tend to settle in specific areas, and geographic variation in risk factors not incorporated into the parent study design.

It is worth noting that total years lived in Marin was found in the parent study to not be a significant risk factor [5]. The identification of localized pockets of elevated risk among long term residents, with immigration from areas outside of Marin of elevated breast cancer incidence does not necessarily contradict this finding. It is not just an issue of how long a woman has lived in Marin County. What appears to influence risk (in addition to those factors identified by Wrensch *et al*. [5]) is (1) where one comes from before moving to Marin and (2) whether or not one resides for a sufficiently long time in specific areas within Marin County.

Several caveats and limitations apply to this study. First, the residential histories were obtained by survey, and their accuracy is unknown. It is likely that the addresses of residences from earlier in a person’s life may be subject to larger recall error, but there is no means of assessing this error. Additional research is needed on recall error in studies of residential mobility.

Second, only ~35% of the residential addresses reported by survey were successfully geocoded, meaning the majority of residential locations from over the participants’ life-course were not accounted for in this study. There were not substantial differences between the numbers of addresses geocoded for the cases and controls, yet the low geocoding rate decreases confidence in the results of the analyses of residential mobility. It would be useful to repeat this study using more complete residential histories. One possibility is commercially available residential histories from, for example, Lexis Nexis, which was demonstrated in one study to provide residential histories with 70% accuracy [14].

Third, the *Ǫ*-statistics employed focus on the cases themselves, evaluating clustering about the cases and not around the controls. This means a study participant can be the center of a cluster of cases only if they are a case themselves. This may not be appropriate for infectious etiologies where people can be carriers but not themselves be infected/symptomatic. Given what is known about the etiology of breast cancer, assessing clustering about cases (and not controls) seems to be a reasonable approach.

Fourth, results from highly significant statistics (*Ǫ*, *Ǫ_i_*, *Ǫ_t_*, and the cardinality of the results from these methods) were used to identify cluster constituents along the lines illustrated in Figure 2. While the burden of evidence demonstrates the persistent, local clusters identified in this study are statistically unusual, a biologically plausible exposure or risk factor has yet to be identified.

Finally, the temporal orientation used in this study is date. Alternative orientations include age, cohort and others [15]. It may be useful to explore hypotheses regarding breast cancer etiology using an age-based temporal orientation to explore developmental windows of vulnerability.

Ignoring statistical issues potentially due to the low fraction (~35%) of addresses that were able to be geocoded, it seems to be an important conclusion from this study that the residential histories of individuals in a community are relevant if not important in determining that community’s composite breast cancer risk and incidence. If this is true, then a question of considerable importance is the magnitude of the excess risk that has been brought into Marin via residential mobility from outside areas (e.g., “imported risk”) and its relative contribution to Marin breast cancer incidence at the time of participants enrollment in the case control study. This important question was addressed in this study by identifying case clustering among long-term residents of Marin (Figure 9), and by identifying outside sources of life-course clustering in Wisconsin, other parts of California, and in the Northeast (Figure 7 and Figure 8). A logical next step would be to perform attributable risk calculations that allocate the observed excess risk to sources within, and outside, of Marin County. This final question is best addressed once accurate sources of residential histories are identified.

## 6. Conclusions

This paper’s merit is three fold. First, it addresses a health issue of some importance: the incidence of breast cancer in Marin County, California. Marin County is unique in that it has among the highest incidence of breast cancer in the United States. This high incidence has persisted for well over a decade, and has been widely studied. While some of the causes underlying the elevated risk have been identified in case-control studies, a large portion of the elevated risk remains unexplained, a problem addressed by this study. Second, this study budgets the unexplained risk geographically and through time, using residential mobility data and accounting for known risk factors and covariates. While this does not of itself evaluate alternative risk factors, the localization of unexplained risk can be a powerful tool in formulating causal hypotheses. The authors identified “movers” and “stayers” among the study participants, and hypothesize immigration from known areas of elevated breast cancer risk (e.g., Long Island for one) as a contributor to the Marin breast cancer excess. Third, this study provides the first comprehensive demonstration of the method of *Ǫ* statistics. The technique has been extensively documented, evaluated in simulation studies, and applied to non-Hodgkin’s lymphoma, testicular cancers, childhood leukemia and bladder cancers. This publication provides a synthesis of the methodological and inferential techniques underpinning *Ǫ* statistic, and then fully demonstrates the approach using the Marin breast cancer data set.

The caveat lies in the geocoding success rate. The original residential history database was comprised of 4,460 address records, an average of 7.81 addresses per participant. Many of these records did not have complete address information. Of these, only 1,878 had complete addresses. Of these, 83% were successfully geocoded, but of the total 4,460 addresses this is only a 38% success rate. This study thus is a clarion call for improved geocoding and address history methodologies, a need that is increasingly recognized in spatial epidemiology. Recent advances in geocoding, geocoding accuracy assessment, and residential address history reconstruction from commercial vendors are just beginning to address this need; a need the authors suggest is one of the more important measurement challenges in spatial epidemiology today.
